# The reported impact of non-communicable disease investment cases in 13 countries

**DOI:** 10.1136/bmjgh-2023-014784

**Published:** 2024-04-10

**Authors:** Giuseppe Troisi, Roy Small, Roman Chestnov, Diana Andreasyan, Henrik Khachatryan, Erwin Arthur Phillips, Taraleen Malcolm, Hero Kol, Nargiza Khodjaeva, Mussie Gebremichael, Addisu Worku Tessema, Asmamaw Bezabeh Workneh, Tamu Davidson, Michelle Harris, Nurgul Ibraeva, Aigul Nurmatova, Aliina Altymysheva, John Juliard Go, Anna Kontsevaya, Krisada Hanbunjerd, Sushera Bunluesin, Olivia Nieveras, Banu Ekinci, Bekir Keskinkiliç, Toker Erguder, Oyoo Charles Akiya, Hafisa Kasule, Aidah Nakanjako, Shukhrat Shukurov, Nazokat Kasymova, Patrick Banda, Ernest Kakoma, Nathan N Bakyaita, Alexey Kulikov, Dudley Tarlton, Nadia Putoud, Scott Chiossi, Douglas Webb, Nicholas Banatvala

**Affiliations:** 1 WHO Regional Office for the Eastern Mediterranean, Cairo, Egypt; 2 United Nations Development Programme, New York, New York, USA; 3 International Telecommunication Union, Geneva, Switzerland; 4 National Institute of Health/National Health Information Analytic Center, Ministry of Health of the Republic of Armenia, Yerevan, Armenia; 5 WHO Armenia, Yerevan, Armenia; 6 Government of Barbados Ministry of Health & Wellness, Saint Michael, Barbados; 7 Pan American Health Organization, Washington, District of Columbia, USA; 8 Department of Preventive Medicine, Royal Government of Cambodia Ministry of Health, Phnom Penh, Cambodia; 9 WHO Cambodia, Phnom Penh, Cambodia; 10 Disease Prevention and Control Directorate, Ethiopia Ministry of Health, Addis Ababa, Ethiopia; 11 Federal Ministry of Health, Addis Ababa, Ethiopia; 12 WHO Country Office for Ethiopia, Addis Ababa, Ethiopia; 13 Ministry of Health and Wellness, Kingston, Jamaica; 14 Ministry of Health and Social Development of the Kyrgyz Republic, Bishkek, Kyrgyzstan; 15 Smoke-free Universities, Bishkek, Kyrgyzstan; 16 WHO Kyrgyzstan, Bishkek, Kyrgyzstan; 17 WHO Philippines, Manila, Philippines; 18 National Research Center for Preventive Medicine, Ministry of Health of the Russian Federation, Moskva, Russian Federation; 19 Division of NCDs, Department of Disease Control, Royal Thai Government Ministry of Public Health, Bangkok, Thailand; 20 WHO Thailand, Bangkok, Thailand; 21 Republic of Türkiye Ministry of Health, Cankaya, Türkiye; 22 WHO Türkiye, Ankara, Türkiye; 23 Department of NCDs, Republic of Uganda Ministry of Health, Kampala, Uganda; 24 WHO Country Office for Uganda, Kampala, Uganda; 25 United Nations Development Programme Uganda, Kampala, Uganda; 26 Healthy Lifestyle and Physical Activity Support Center, Ministry of Health of the Republic of Uzbekistan, Tashkent, Uzbekistan; 27 WHO Uzbekistan, Tashkent, Uzbekistan; 28 Zambia Ministry of Health, Lusaka, Zambia; 29 WHO, Lusaka, Zambia; 30 United Nations Inter-Agency Task Force on the Prevention and Control of NCDs, WHO, Geneva, Switzerland; 31 Health and Development, United Nations Development Programme, Geneva, Switzerland; 32 The Global Fund to Fight AIDS, Tuberculosis and Malaria, Grand-Saconnex, Switzerland; 33 UNDP, Tblisi, Georgia

**Keywords:** global health, health economics, health education and promotion, health policy, prevention strategies

## Abstract

Non-communicable diseases (NCDs) are a leading health and development challenge worldwide. Since 2015, WHO and the United Nations Development Programme have provided support to governments to develop national NCD investment cases to describe the socioeconomic dimensions of NCDs. To assess the impact of the investment cases, semistructured interviews and a structured process for gathering written feedback were conducted between July and October 2022 with key informants in 13 countries who had developed a national NCD investment case between 2015 and 2020. Investment cases describe: (1) the social and economic costs of NCDs, including their distribution and projections over time; (2) priority areas for scaled up action; (3) the cost and returns from investing in WHO-recommended measures to prevent and manage NCDs; and (4) the political dimensions of NCD responses. While no country had implemented all the recommendations set out in their investment case reports, actions and policy changes attributable to the investment cases were identified, across (1) governance; (2) financing; and (3) health service access and delivery. The pathways of these changes included: (1) stronger collaboration across government ministries and partners; (2) advocacy for NCD prevention and control; (3) grounding efforts in nationally owned data and evidence; (4) developing mutually embraced ‘language’ across health and finance; and (5) elevating the priority accorded to NCDs, by framing action as an investment rather than a cost. The assessment also identified barriers to progress on the investment case implementation, including the influence of some private sector entities on sectors other than health, the impact of the COVID-19 pandemic, and changes in senior political and technical government officials. The results suggest that national NCD investment cases can significantly contribute to catalysing the prevention and control of NCDs through strengthening governance, financing, and health service access and delivery.

**WHAT IS ALREADY KNOWN ON THIS TOPIC**

- Non-communicable diseases (NCDs) are a leading health and development challenge worldwide, with low-income and middle-income countries facing large burdens.
- National NCD investment cases have provided compelling data on the socioeconomic aspects of NCDs and the return on investment (ROI) in priority interventions.
- There has been no systematic assessment of the impact of these investment cases on policymaking.

**WHAT THIS STUDY ADDS**

- In countries where NCD investment cases have been undertaken, there is evidence of actions and policy changes that can be at least partly attributed to an investment case.
- ROI analyses played a key role in driving policy changes, with policy packages projecting high ROI receiving significant attention.
- Commercial influence on sectors other than health represents a major barrier to investment case implementation.

**HOW THIS STUDY MIGHT AFFECT RESEARCH, PRACTICE OR POLICY**

- National NCD investment cases provide evidence and advocacy that can catalyse stronger NCD responses through improved governance, financing, and health service access and delivery.
- The investment cases provide opportunities for cross-government action, and allow NCDs to be framed as an investment rather than a cost.
- There is a need to develop more detailed economic analysis on issues closely tied with commercial determinants of health, such as nutrition, alcohol and health taxes.

## Introduction

Non-communicable diseases (NCDs)—including cancers, diabetes, cardiovascular disease and chronic respiratory disease—are a leading health and development challenge worldwide. NCDs kill 41 million people each year, comprising 74% of all deaths globally, with 17 million people dying prematurely from an NCD (before age 70 years) each year, of which 86% of these deaths are in low-income and middle-income countries.^1^ In addition to their health impact, NCDs detract from economies through productivity losses comprised of early workforce dropout, missed work (absenteeism) or reduced performance at work (presenteeism).^2 3^

The main behavioural and environmental risk factors for NCDs are tobacco use, harmful use of alcohol, unhealthy diet, physical inactivity and air pollution. Cost-effective and evidence-based interventions exist for preventing and controlling NCDs.^4^ However, governments need to determine the cost-effectiveness of these interventions in their particular context, as well as non-financial considerations such as implementation capacity, feasibility and impact on health equity.^5^

Since 2015, WHO and the United Nations Development Programme have provided support to governments to develop national NCD investment cases that assess the social and economic costs of NCDs, including their distribution and projections over time; identify priorities for scaled up action; and estimate the cost and returns from investing in WHO-recommended measures to prevent and manage NCDs.^6^ The cases include an institutional and context analysis (ICA) which explores the political dimensions of NCD responses to inform pathways to implementation.^7^

The investment cases have provided compelling data on the socioeconomic impact of NCDs and the return on investment (ROI) from implementing interventions to prevent and control NCDs.^8 9^ They have also provided context-specific governance and financing recommendations, for example, around improving multisectoral planning and coordination and/or strengthening health taxes as a means of sustainable domestic financing. However, there has been no systematic attempt to assess the impact of these investment cases on policymaking. The present study therefore aims to do this.

## Methods

16 countries that had undertaken NCD investment cases between 2015 and 2020 were invited to participate in the study. 13 agreed to participate. They were: Armenia, Barbados, Cambodia, Ethiopia, Jamaica, Kyrgyzstan, Russian Federation, Philippines, Thailand, Türkiye, Uganda, Uzbekistan and Zambia.

### Identifying impacts among the countries receiving investment cases

To assess the impact of the investment cases, semistructured interviews and a structured process for obtaining written feedback were conducted with a total of 30 key informants across the 13 countries between July and October 2022. Particular attention was given to understanding impacts beyond the health sector (Box 1).

Box 1 **Areas of potential impact explored in the interviews**

1. How the investment case was used.

2. Impact of the investment case on:

- Non-communicable disease (NCD) prevention and control policies and actions.
- NCD funding.
- NCD planning, coordination and policy coherence including around commercial determinants of health.
- Adoption and ownership of the NCD response in sectors other than health.
- Ownership of the NCD response beyond government.

3. Areas where further attention is required to drive action.

4. Any other points/observations.

In the first instance, interviews were undertaken with 14 United Nations staff who were involved in the development of the investment case, supporting its implementation and/or tracking progress. Following the interviews, summaries were drafted on the impact of the investment cases for each country. These were then shared with those interviewed, with a request that the summaries be shared more widely with individuals across different government departments for further reflection and input. Written inputs into the summaries were received from technical experts across government from each of the countries in the study. These included 15 ministries of health focal points and 1 representative from academia, all of whom are coauthors on this paper.

The summaries were then reviewed together, and their impacts were grouped into: (1) governance, including laws, policies, plans, coordination and public communications; (2) financing, including budget allocation, leveraging additional partnership support, and health taxes; and (3) health service access and delivery, including health system strengthening, universal health coverage and service provision. These results were then shared once again with key informants for final inputs.

## Results

### Impacts on governance, health service access and delivery, and financing

Across the 13 countries, 47 actions and/or policy changes were identified as being attributable in whole or in part to the NCD investment cases. These are summarised in table 1. Each country identified at least one action that had resulted from the investment case. Governance was the area most frequently identified, followed by financing and then health service access and delivery. For NCD risk factors, NCD investment cases had the greatest impact on action related to tobacco control (n=10; six countries) followed by reducing unhealthy diet (n=6; six countries), reduction in harmful use of alcohol (n=4; three countries) and improving levels of physical activity (n=3; three countries). The full list of recorded actions and policy changes is available in online supplemental table 1.

10.1136/bmjgh-2023-014784.supp1 Supplementary data

**Table 1:** Summary of advancements attributable wholly or in part to investment cases

Country	Year investment case initiated	Action	Impact area
Armenia	2018	Adopted law on tobacco use reduction (2020)	Governance
		Increased budget allocated for NCD (2021)	Financing
Barbados	2015	Introduced sugar-sweetened beverages tax (2015, increased in 2022)	Governance, financing
		Dedicated budget to NCD, including by nominating a minister for NCDs	Governance, financing
		Implemented cardiovascular risk reduction initiatives and strengthened capacity of community health workers	Health service access and delivery
		Continued multisectoral coordination for NCD	Governance
Cambodia	2018	Endorsed smoke-free environment in tourism (2022)	Governance
		Establishment of working group on tobacco (2022)	Governance
		Developed *Strategic Plan for Tobacco Control* (2021)	Governance
		Developed action plan for salt reduction (2021)	Governance
		Developed national strategic plan on NCDs (2022)	Governance
		Prioritised NCD interventions in primary care (2022)	Financing
Ethiopia	2018	Adopted strategic action plan on NCDs (2020)	Governance
		Conducted training exercises for NCD management (ongoing)	Health service access and delivery
		Reformed tobacco and alcohol tax structure (2020)	Governance, financing
		Adopted ban on alcohol advertisement (2020)	Governance
		Launched salt reduction media campaign (2021)	Governance
		Increased spending for NCDs (2020)	Financing
		Established NCD multisectoral committee (2020)	Governance
Jamaica	2017	Implemented health system strengthening programme, with a focus on NCD (2019)	Health service access and delivery, financing
		Carried out awareness-raising campaign on physical activity and nutrition (2018/2019/2022)	Governance
		Increased funding to NCD during, and in response to, the COVID-19 pandemic (2020)	Financing
		Reactivated National Committee on NCDs (2021)	Governance
		Policy shift to address NCD risk factors (*Taking Responsibility* Programme) (2018)	Governance
Kyrgyzstan	2016	Adopted the law on tobacco consumption (2021)	Governance
		Implemented smoke-free policies in parks and playgrounds across municipalities (2021)	Governance
		Organised the World Nomad Games in a smoke-free environment (2018)	Governance
Philippines	2018	Implemented excise taxes on tobacco and alcohol and earmarked revenues to fund a universal health coverage scheme (2019)	Governance, financing, health service access and delivery
		Adopted policy on elimination of trans-fats (2021)	Governance
		Launched health promotion strategy 2030 (2021)	Governance
Russian Federation	2019	Adopted federal law on tobacco consumption (2021)	Governance
		Allocated budget for the implementation of health promotion project, including on communication campaigns on NCD risk factors (2020)	Financing
Thailand	2020	Adopted national plan on NCD (2022)	Governance
		Extended the term of multisectoral committee on NCDs (2023)	Governance
Türkiye	2017	Increased coverage of NCD-related clinical services at primary care (2022)	Health service access and delivery, financing
		Launched campaigns on salt reduction and early detection of kidney disease (2018)	Governance
		Established intersectoral working group on NCDs (2018)	Governance
Uganda	2019	Integrated NCDs into HIV/AIDS service delivery points, by leveraging US$4.5 million PEPFAR donation (2022)	Health service access and delivery, financing
		Drafted *NCD Multisectoral Strategy*	Governance
		Established national NCD multisectoral committee chaired by the prime minister (2022)	Governance
Uzbekistan	2017	Issued presidential decree to encourage physical activity (2020) and organised public communication to promote physical activity and healthy lifestyle (2020–2022)	Governance
		Launched national NCD strategy (2018)	Governance
		Established the Healthy Lifestyle Center (2018) and intersectoral commission on disease prevention and public health (2020)	Governance
Zambia	2017	Catalysed the 2022 NCDI Poverty Commission Report	Governance
		Catalysed resource mobilisation for NCDs from the Center for Infectious Disease Research	Financing, health service access and delivery
		Developed multisectoral NCD strategic plan (2022)	Governance, financing

NCD, non-communicable disease; NCDI, National Noncommunicable Disease and Injury; PEPFAR, President's Emergency Plan for AIDS Relief.

Investment case ROI figures for the 13 countries are shown in table 2. Care should be taken in comparing ROIs across countries as the investment case methodology varied slightly for each country, for example, because of differences in the package of interventions assessed or data available. Further details are available on the United Nations NCD Task Force website.^10^

**Table 2:** ROI figures from national NCD investment cases (over a 15-year period)

Country	Tobacco	Alcohol	Salt	Physical activity	Clinical intervention package for CVD and diabetes
Armenia	14.5	4.1	14.3	4.4	0.3
Barbados*	Package warnings: 38 Advertising bans: 26 Cessation programmes: 11	NA	NA	NA	CVD Combination drug therapy for those at CVD risk: 15.5 Drug therapy for those with hypertension: 0.7 Drug therapy for those with high cholesterol: 1.9 Aspirin post-acute stroke: 15.5 Combination drug therapy for those with IHD: 31 Combination drug therapy for those with stroke: 37.5 Diabetes Standard glycaemic control: 0.3 Intensive glycaemic control: 1.1
Cambodia	10.7	5.0	9.6	10.0	0.2
Ethiopia	3.1	1.4	3.3	1.8	0.1
Jamaica	5.37	1.86	NA	NA	CVD: 1.9 Diabetes: 2.1
Kyrgyzstan	3.8	NA	12.3	3.6	CVD: 0.01
Philippines	8.8	7.0	29.9	12.7	0.1
Russian Federation†	NA	NA	NA	NA	NA
Thailand	2.53	2.28	10.26	1.82	1.25
Türkiye	5.0	0.6	88.0	2.3	4.3
Uganda†	NA	NA	NA	NA	NA
Uzbekistan	13.0	1.9	64.8	9.6	0.3
Zambia	5.3	1.3	NA	4.5	4.6

*The capacity to calculate ROI for a number of policy options was not available at the time of undertaking the analysis in Barbados. In addition, ROI analysis was broken down by specific interventions, rather than by intervention package.

†ROI figures not publicly available at the time of writing.

CVD, cardiovascular disease; IHD, ischaemic heart disease; NA, not available; NCD, non-communicable disease; ROI, return on investment.

According to key informants, the ROIs in the individual investment cases played a role in driving policy changes at the national level. In general, policy packages related to tobacco control and salt reduction had higher ROI, and as a result, interviews suggested that recommendations for tobacco control and salt reduction received significant attention.

Nevertheless, impact areas were not solely driven by ROI considerations. Clinical interventions tended to have lower ROI in the investment cases, yet nonetheless six countries attributed improvements in strengthening NCD management to the investment case. Key informants noted the need to prioritise action among those who required treatment, and that clinical interventions usually project to save the most lives, as factors.

Informants also described the impact that the investment cases had on facilitating dialogue across sectors and with partners, in raising awareness on NCDs as both a health and development priority, and encouraging joint action. In particular, the results of the ICA and recommendations in the investment case reports^10^ informed opportunities for cross-government action to tackle NCDs. In general, a main observation emphasised during the interviews was that investment cases are instrumental in framing NCD action as an investment, rather than a cost (table 3).

**Table 3:** Key informant perspectives on the value of the investment cases

Country	Quote
Armenia	*The NCD investment case was instrumental in informing the advocacy strategy of the Ministry of Health to adopt the Tobacco Control Law in 2020. Staggering results from the economic analysis made the Government, and especially the Ministry of Finance, consider health as an investment, not a cost*.
Barbados	*The investment case has helped to promote the idea that allocating resources to NCD prevention and control is an investment rather than cost*.
Cambodia	*The NCD investment case is considered a valuable source of data not only for promoting action on NCDs, but also for planning of broader public health interventions*.
Ethiopia	*The investment case has been widely used as an advocacy tool since its launch in 2019, as well as a background paper guiding NCD action. For instance, it contributed to building the argument to increase the budget of the NCD team within the Ministry of Health*. *Findings from the investment case supported collaboration between Ministries of Health and Finance around the taxation on health harming products, that is, tobacco and alcohol. It was also used to support the ban on alcohol advertisement*.
Jamaica	*The investment case showed a significantly high ROI for the tobacco control policy package, generating political appetite for comprehensive tobacco law(s). Jamaica is currently debating a comprehensive tobacco control law in Parliament, which includes adequate taxation*. *Data around the NCD burden, which is specific to the national context, rather than aggregated regional or global data, were picked up very quickly by the Ministry of Health to guide prioritization of funding towards national health system strengthening programmes particularly focused on the control of NCDs*. *The NCD investment case provided the rationale to increase funding to the NCD response*.
Kyrgyzstan	*The investment case report supported the MOH in building the argument to increase excise taxes on tobacco products, by disseminating solid economic data and establishing an effective dialogue with the Ministry of Finance*. *Tobacco control initiatives and interventions recommended in the investment case report were moved forward also by government sectors other than health. Municipalities have implemented smoke-free parks and playgrounds, and academia is being sensitized to advocate for smoke-free university campuses*.
Philippines	*The investment case process was an opportunity to bring NCDs higher on the UN agenda: and NCD-related targets are included in the UN Sustainable Development Cooperation Framework of the Philippines*. *In 2020, during the COVID-19 pandemic the Government tried to address the disruption of NCD-related services, by promoting telemedicine. However, the pandemic proved a challenge to following up on the investment case recommendations except for developing and disseminating public health messaging around NCD risk factors*.
Russian Federation	*The national stakeholder mapping and the relevant analysis contained in the ICA assisted the MOH convening a consultation to develop and adopt a new anti-tobacco legislation*. *The Ministry of Health used economic results from the investment case to convince the Ministry of Finance to allocate adequate resources for a Federal Project on Public Health and Risk Factor Prevention(…). As a result of the regional public health programme, awareness raising campaigns were organized, regional and municipal public health programmes were introduced*. *The investment case provided the Ministry of Health and the Ministry of Finance with common vocabulary*.
Thailand	*The investment case report has been used to develop key advocacy messages addressed to sectors other than health, including awareness campaigns jointly developed by WHO and UNDP*.
Türkiye	*The investment case provided the argument to strengthen delivery of NCD-services in PHC. This resulted in family physicians and family health workers receiving positive performance payments which will be increased depending on screening and monitoring rates*. *The NCD investment case helped the Ministry of Health build a solid economic argument to advocate with the Ministry of Finance for increased budget to implement NCD-related clinical interventions at the primary healthcare level*.
Uganda	*Academia and civil society organizations not only participated in the development of the NCD investment case, but contributed to supporting its dissemination, as well as the implementation of interventions. Civil society and other implementing partners used data from the NCD investment case to plan the roll out of HIV/NCD integration into PEPFAR 2022 operational plan implementation*.
Uzbekistan	*The NCD investment case was a real eye-opener for the Government; economic data convinced ministries other than health that it was necessary to tackle NCDs*. *The investment case helped to secure political commitment to tackle NCDs, from the President and the Prime Minister. The President’s Decree (December 2018) launched the National NCD Strategy 2019–2022, which entailed a re-organization of the structure of the Ministry of Health and the establishment of the Healthy Lifestyle Center with its regional units across the country, as well as the creation of an intersectoral coordination commission for disease prevention and public health, with a dedicated budge, In November 2020*. *The national media has promoted messages around NCD prevention and control, including data extrapolated from the investment case*.
Zambia	*The NCD investment case report was integrated into the Zambia NCDI Poverty Commission Report 2022, the findings of which contributed to securing financial support from the Center for Infectious Disease Research towards implementing the WHO Package of Essential NCD interventions*. *The NCD investment case (1) facilitated advocacy with policy makers on promotion of healthy lifestyles in workplaces, (2) leveraged partnerships to support the PHC package towards the attainment of UHC, (3) elevated the priority of NCDs, by framing action as an investment rather than a cost, and (4) findings were incorporated in the National Health Strategic plan 2022–2026 and the NCD Strategic plan draft*.

ICA, institutional and context analysis; MOH, Ministry of Health; NCD, non-communicable disease; NCDI, National Noncommunicable Disease and Injury; PEPFAR, President's Emergency Plan for AIDS Relief; PHC, primary healthcare; ROI, return on investment; UHC, universal health coverage; UN, United Nations; UNDP, United Nations Development Programme.

No country in this study has implemented all the modelled interventions and recommendations set out in their investment case—and some countries have only implemented a small number. The interviews and their reviews identified barriers to greater progress. A main obstacle was opposition from certain private sector industries and their influence on ministries outside the health sector, such as ministries of agriculture, commerce, finance, labour, trade and transport. A second barrier identified was that the COVID-19 pandemic diverted attention from NCDs.^11^ A third barrier was changes in political and technical officials, including ministers and high-level government officials. This made needed commitment from sectors other than health difficult to secure and sustain in some circumstances. Funding was not raised by those interviewed as a major barrier to implementing the recommendations of the investment case reports.

## Discussion

Although the NCD investment case approach has limitations that have been described elsewhere,^12^ these are unlikely to have affected their impact as a tool to encourage policy change, as all investment case findings were validated and reports cleared by relevant national authorities. However, the methods described in this paper have notable limitations. First, despite the responses from those interviewed and those reviewing the results, it is important to recognise the range of other NCD prevention and control efforts in each of the 13 countries before, during and after the investment cases, making it difficult to determine the precise attribution or contribution from the cases. Second, there is potential for interviewees to have provided overly positive reporting. That may be because they were directly involved in the work and/or wish to maintain good standing with partners and funders. Third, there was no assessment of changes in policy among a comparative set of countries that had not undertaken investment cases. Finally, despite follow-up, 3 of the 16 countries invited to participate did not respond. These may be countries where the impact was less or even negative.

This study suggests that national NCD investment cases are effective at catalysing the prevention and control of NCDs through strengthening governance, financing, and health service access and delivery. Governance impacts spanned new or stronger laws and/or policies, improved plans and coordination mechanisms, and scaled up public communications. Financing impacts included budgetary allocations to NCDs, use of health taxes including mobilising domestic resources and leveraging development assistance funding. Many of the impacts yielded broader health and development benefits, for example, progress towards universal health coverage.

Results also highlighted elements that enabled the changes across governance, financing, and health service access and delivery. These included: (1) strengthening collaboration across government ministries and partners; (2) strengthening advocacy for NCD prevention and control; (3) grounding efforts in nationally owned evidence; (4) developing shared ‘language’ across health and finance; and (5) elevating the priority of NCDs, by framing action as an investment rather than a cost. Informants stressed that the impacts stemmed from not just the investment case report but also from the inclusive and participatory process of developing it. The ICAs were crucial to this.

That tobacco control was the area of greatest impact coheres with it typically having the highest ROI across the investment cases, a consideration reported by key informants. Another explanation for this not explicitly mentioned may be that all participating countries were Parties to the WHO Framework Convention on Tobacco Control, a legally binding international treaty. Armenia and Cambodia were also supported by tobacco control-specific investment cases through another United Nations project.^13 14^ At the same time, the countries did not just pursue the highest ROIs. For example, clinical interventions received strong attention despite typically projecting lower ROIs. In addition to the reasons provided by key informants, a factor may be that countries are encouraged to implement the investment case recommendations in full, seeing prevention and treatment as mutually reinforcing and both critical to the right to health.

The reported effect of commercial influence on government sectors other than health as a barrier to fuller investment case implementation suggests that technical support on whole-of-government responses, including on policy coherence and prevention of industry interference in policymaking, would be useful complements to investment cases. In addition, it may be useful to develop a more detailed economic analysis on specific issues closely tied with commercial influence, such as nutrition, alcohol and health taxes, just like what has been done with tobacco control, using tobacco control-specific investment cases. That the COVID-19 pandemic diverted attention from NCD action reinforces the urgency of ensuring NCDs are included in global health security and universal health coverage. Chronic and infectious diseases can be mutually exacerbating, for example, NCDs and their risk factors are associated with worse COVID-19 outcomes, and SARS-CoV-2 infection is associated with increases in NCDs.^15^ Finally, to counter waning commitment to tackle NCDs as a result of changes in ministers and government officials, the investment cases and their recommendations may need refreshing and/or strategic ‘relaunch’ over time.

## Data Availability

All data relevant to the study are included in the article or uploaded as supplemental information.
