# Supplementary material for: The reported impact of non-communicable disease investment cases in 13 countries
Source: BMJ Glob Health. 2024 Apr 10;9(4):e014784. doi: 10.1136/bmjgh-2023-014784 (PMC11015227; doi:10.1136/bmjgh-2023-014784)
Supplement: Supplementary data [file bmjgh-2023-014784supp001.pdf]

BMJ Publishing Group Limited (BMJ) disclaims all liability and responsibility arising from any reliance placed on this supplemental material which has been supplied by the author(s)

Supplementary Table 1. Advancements attributable wholly or in part to investment cases

Country	Year investment case initiated	Action	Impact area
**Armenia**	2018	Adopted the law on *Reduction and Prevention of the Damage Caused by the Use of Tobacco Products and Substitutions for Them* (2020).	Governance
		Increased budget allocated for NCD prevention and control in the context of the *National Healthy Lifestyle Strategy* by 10% compared to the 2020 budget (2021).	Financing
**Barbados**	2015	Introduced a 10% sugar-sweetened beverages tax in 2015, which was increased to 20% in 2022.	Governance<br><br>Financing
		Dedicated budget to NCD prevention and control, including by nominating a Minister for NCDs, within the Ministry of Health and Wellness.	Governance<br><br>Financing
		Implemented cardiovascular risk reduction initiatives in collaboration with hospitals, and strengthened capacity for the NCD response among community health workers.	Health service access and delivery
		Continued multisectoral coordination through the NCD Commission (started in 2007), National Workplace Wellness Committee (started in 2019), Wellness Task Force (started in 2018).	Governance
**Cambodia**	2018	Endorsed Standard Operating Procedure for smoke-free environment in tourism (2022).	Governance
		Prakas (official proclamation) on the establishment of the Working Group on Tobacco Taxation and Control of Illicit Trade in tobacco products (WGTT) issued by the Deputy Prime Minister of Economy and Finance (2022).	Governance
		Developed the *Guidelines for Smoke-Free Tourism City* (2022).	Governance
		Developed the *National Action Plan for Salt Reduction 2021-2027* (2021).	Governance
		Developed the *Strategic Plan for Tobacco Control 2021-2026* (2021).	Governance
		Developed the *National Strategic Plan on NCDs prevention and control 2022-2030* (2022).	Governance
		Prioritized NCD interventions in primary healthcare, through the World Bank project on *Health Equity and Quality Improvement II* (H-EQIPII, 2022).	Financing
**Ethiopia**	2018	Adopted *National Strategic Action Plan for the Prevention and Control of NCDs 2020/21-2024/25* (2020).	Governance

		Conducted training exercises, including *Effective Management and Proper Utilization of Pharmaceuticals used for Non-Communicable Diseases Management for Health Facility Pharmacy Professionals* and *Hypertension Evidence-based Treatment Protocol for Primary Health Care and General Hospital settings in Ethiopia* (ongoing).	Health service access and delivery
		Reformed the tobacco and alcohol tax structure, including by introducing a mixed-excise system on cigarettes, thus increasing the tax share of the retail price (2020).	Governance<br><br>Financing
		Adopted complete ban on alcohol advertisement (2020).	Governance
		Launched national salt reduction media campaign (2021).	Governance
		Increased spending for NCDs as a percentage of total health expenditure from 11% in 2016 to 25% (2020).	Financing
		Established a national NCD multisectoral committee chaired by the Ministry of Health, with co-deputies from House of People's Representatives and Ethiopian Environmental Protection Agency (2020).	Governance
		Implemented *National Health System Strengthening Programme*, with a focus on NCD prevention and control, including by securing US$ 100 million in loans from the Inter-American Development Bank (2019).	Health service access and delivery<br><br>Financing
		Carried out *Jamaica Moves*, a nation-wide awareness raising campaign on physical activity and nutrition (2018-2019-2022).	Governance
**Jamaica**	2017	Increased funding to NCD prevention and control during, and in response to, the COVID-19 pandemic (2020).	Financing
		Reactivated the National Multisectoral Committee on NCDs, originally established in 2011 (2021).	Governance
		Informed Jamaica's policy shift to ensure a comprehensive approach to NCD risk factors, through the *Taking Responsibility* programme (2018).	Governance
		Adopted the law *On protecting the health of citizens of the Kyrgyz Republic from the consequences of tobacco consumption, nicotine and exposure to second hand tobacco smoke and aerosol* (2021).	Governance
**Kyrgyzstan**	2016	Implemented smoke-free policies in parks and playgrounds across municipalities (2021).	Governance
		Organized the World Nomad Games in a smoke-free environment, through collaboration between health and sport sectors (2018).	Governance
**Philippines**	2018	Implemented excise taxes on tobacco and alcohol, with revenue earmarked to fund a national universal health	Governance

		coverage scheme, which extends provision of NCD-related services free of charge at the PHC level (2019).	Financing<br><br>Health service access and delivery
		Adopted the *National Policy on the Elimination of Industrially Produced Trans-Fatty Acids for the Prevention and Control of Non-Communicable Diseases* (2021).	Governance
		Launched the *Health Promotion Framework Strategy 2030* providing a roadmap for implementation of NCD-related interventions (2021).	Governance
**Russian Federation**	2019	Adopted *Federal Law On Amendments to Certain Legislative Acts of the Russian Federation on the Protection of Citizens' Health from the Consequences of Consuming Nicotine-Containing Products*, following multisectoral dialogues of the working group on tobacco control (2021).	Governance
		Advocated Ministry of Finance to allocate adequate budget for the implementation of National Project on Demography and National Project on Healthcare. Funds were allocated on federal public communication campaigns on NCD risk factors (2020).	Financing
**Thailand**	2020	Adopted the *National Plan on NCD Prevention and Control 2023-2027* (2022).	Governance
		Developed terms of reference for the extension of the UN Thematic Working Group on Noncommunicable Diseases in Thailand, after a first three-year term (2018-2020) (2023).	Governance
**Türkiye**	2017	Increased coverage of NCD-related clinical services at the PHC level, in the context of the *National Health System Strengthening Programme*, following negotiation with the Ministry of Finance to dedicate relevant budget (2022).	Health service access and delivery<br><br>Financing
		Launched nation-wide campaigns on salt reduction and early detection of kidney disease (2018).	Governance
		Established intersectoral working group on NCDs, hosted in the MOH (2018).	Governance
**Uganda**	2019	Facilitated advocacy and partnership engagement between MOH, Parliament of Uganda, PEPFAR, WHO and UNDP to integrate NCDs into HIV/AIDS service delivery points. USD 4.5 million was allocated for hypertension, diabetes and cervical cancer screening and management in the Country Operational Plan for 2022 (COP 22).	Health service access and delivery<br><br>Financing
		Drafted *NCD Multisectoral Strategy*, currently under the review of the NCD Technical Working Group.	Governance

		Established national NCD multisectoral committee chaired by the Prime Minister (2022).	Governance
**Uzbekistan**	2017	Issued the Decree of the President *On measures for the widespread introduction of a healthy lifestyle and the further development of mass sports* (2020), and organized public communication to promote physical activity and healthy lifestyle (2020-2022).	Governance
		Launched the *National NCD Strategy 2019-2022* with the President's Decree *On measures to prevent non-communicable diseases, support a healthy lifestyle and increase the level of physical activity of the population* (2018).	Governance
		Established the Healthy Lifestyle Center with its Regional Units, under the MOH, in accordance with the President's Decree *On measures to prevent non-communicable diseases, support a healthy lifestyle and increase the level of physical activity of the population* (2018), as well as a intersectoral coordination commission on disease prevention and public health under the Government, established in accordance with the President's Decree *On additional measures to ensure public health by further improving the efficiency of medical prevention work* (2020).	Governance
**Zambia**	2017	Catalysed the 2022 NCDI Poverty Commission Report	Governance
		Catalysed resource mobilization from the Center for Infectious Disease Research in Zambia to improve management of severe NCD in primary health care services.	Financing<br><br>Health service access and delivery
		Leveraged partners support for the development of the *National Multisectoral NCD Strategic Plan 2022-2026*.	Governance<br><br>Financing
